# Sex differences in acute axon degeneration after traumatic brain injury in swine

**DOI:** 10.1002/alz70855_103002

**Published:** 2025-12-23

**Authors:** Hailong Song, Yue Qiu, Jean‐Pierre Dolle, D. Kacy Cullen, William Stewart, Douglas H Smith

**Affiliations:** ^1^ University of Pennsylvania, Philadelphia, PA, USA; ^2^ University of Glasgow, Glasgow, United Kingdom; ^3^ Queen Elizabeth University Hospital, Glasgow, United Kingdom

## Abstract

**Background:**

Although human females appear at a higher risk of traumatic brain injury (TBI) and suffer worse outcomes than males, underlying mechanisms remain unclear. With increasing recognition that damage to white matter axons is a key pathologic substrate of TBI, we used a clinically relevant swine TBI model to explore potential sex differences in the extent of axonal pathologies in relation to axon size. Since TBI is one major risk factor for the development of Alzheimer's disease (AD), we further examined sex differences in the accumulation of amyloid precursor protein (APP) and amyloid‐beta (Aβ) in damaged and degenerating axons.

**Method:**

We used a well‐characterized swine TBI model, which is scaled to mimic head rotational acceleration kinematics of human TBI. Using immunohistochemical staining, we first examined the extent of APP+ axonal pathology, which arises due to microtubule damage and impaired axonal transport, and compared the accumulation of Aβ in damaged axons between sexes. In addition, we assessed the extent of loss of the predominant axonal sodium channel (NaCh) that populates the nodes of Ranvier, Nav1.6. Moreover, using transmission electron microscopy, we evaluated differences in axonal diameter between female and males with no injury and changes in the average caliber of white matter axons after injury between the sexes.

**Result:**

At 24 hours post‐injury, female swine displayed a greater number of swollen axonal profiles accumulating APP and Aβ, particularly at brain white matter. Females also presented more widespread loss of NaChs than males. Axon degeneration for both sexes appeared to be related to individual axon architecture, reflected by a selective loss of small caliber axons after concussion. However, female brains had a higher percentage of small caliber axons, leading to more extensive axon loss after injury compared to males.

**Conclusion:**

These data identify sex differences in the extent of axonal pathologies in brain white matter acutely after TBI, which appears related to sex differences in axon diameter. Notably, these findings further shed light on insights that damaged and degenerating axons following TBI may provide a rich source of APP/Aβ as pathologic substrate contributing to the development of AD and its sex differences.

